# Identification of hepatocellular carcinoma subtypes based on PcG-related genes and biological relevance with cancer cells

**DOI:** 10.1186/s13148-022-01393-6

**Published:** 2022-12-24

**Authors:** Yunong Fu, Kaibo Yang, Kunjin Wu, Hai Wang, Qinglin Li, Fengping Zhang, Kun Yang, Qing Yao, Xiaohua Ma, Yujie Deng, Jingyao Zhang, Chang Liu, Kai Qu

**Affiliations:** 1 Department of Hepatobiliary Surgery, The First Affiliated Hospital of Xi’an Jiaotong University, Xi’an, 710061 Shaanxi China; 2 Department of Surgical Intensive Care Units, The First Affiliated Hospital of Xi’an Jiaotong University, Xi’an, 710061 Shaanxi China; 3 Department of Geriatrics, Xijing Hospital, Fourth Military Medical University, Xi’an, Shaanxi China

**Keywords:** Hepatocellular carcinoma, Polycomb group proteins, Cancer subtype, Chemotherapy sensitivity, Cell metabolism

## Abstract

**Background:**

Hepatocellular carcinoma (HCC) is an extensive heterogeneous disease where epigenetic factors contribute to its pathogenesis. Polycomb group (PcG) proteins are a group of subunits constituting various macro-molecular machines to regulate the epigenetic landscape, which contributes to cancer phenotype and has the potential to develop a molecular classification of HCC.

**Results:**

Here, based on multi-omics data analysis of DNA methylation, mRNA expression, and copy number of PcG-related genes, we established an epigenetic classification system of HCC, which divides the HCC patients into two subgroups with significantly different outcomes. Comparing these two epigenetic subgroups, we identified different metabolic features, which were related to epigenetic regulation of polycomb-repressive complex 1/2 (PRC1/2). Furthermore, we experimentally proved that inhibition of PcG complexes enhanced the lipid metabolism and reduced the capacity of HCC cells against glucose shortage. In addition, we validated the low chemotherapy sensitivity of HCC in Group A and found inhibition of PRC1/2 promoted HCC cells’ sensitivity to oxaliplatin in vitro and in vivo. Finally, we found that aberrant upregulation of CBX2 in Group A and upregulation of CBX2 were associated with poor prognosis in HCC patients. Furthermore, we found that manipulation of CBX2 affected the levels of H3K27me3 and H2AK119ub.

**Contributions:**

Our study provided a novel molecular classification system based on PcG-related genes data and experimentally validated the biological features of HCC in two subgroups. Our founding supported the polycomb complex targeting strategy to inhibit HCC progression where CBX2 could be a feasible therapeutic target.

**Supplementary Information:**

The online version contains supplementary material available at 10.1186/s13148-022-01393-6.

## Background

Hepatocellular carcinoma (HCC) is one of the most common digestive malignancies in the world [1]. HCC is widely accepted to have heterogeneous genomic profiles contributing to its complexity in diagnosis and management [2]. There is an urgent need to employ integrative multi-omics profiling data to elucidate hepatocarcinogenesis and identify molecular mechanisms underlying the complex clinical features of HCC [3].

Epigenetic alteration is expected to make a significant contribution to heterogeneous features of HCC. Accumulating evidence indicates that polycomb group (PcG) complexes are the master epigenetic regulators which conduct transcriptional repression of target genes via modifying the chromatin [4]. In general, PcG complexes are divided into four types, including polycomb-repressive complex1/ 2 (PRC1/2), polycomb-repressive deubiquitylase (PR-DUB), and pho-repressive complex (PhoRC), to control a different modification of histone in the nucleus [5–8]. To constitute PcG complexes, numerous PcG subunits dynamically associate with each other and form a complicated molecular network in the cellular process [9–11]. Furthermore, the cross-talk between different types of PcG complex, such as the hierarchical recruitment model coordinated by PRC1 and PRC2, develops another dimension to enrich the interaction pattern of PcG protein [12].

Structurally, compared to PR-DUB and PhoRC, PRC1/2 both have more alternative subunits and higher diversity, which makes their function complicated [13]. Canonical PRC1 constitutes four core subunits: PCGF2/4 (Polycomb group RING finger proteins), CBX2/4/6/8 (Chromobox), RING1A/B (Ring finger protein), and PHC1/2/3 (Polyhomeotic homolog), while non-canonical PRC1 has other alternative subunits like E2F6 and YAF2 besides PCGF1-6, but is independent of CBX family. Mammalian PRC2 is formed by three core subunits, EZH1/2 (enhancer of zeste 1/2), SUZ12 (suppressor of zeste 12), and EED (embryonic ectoderm development) [14]. Importantly, PRC2 has several facultative subunits, which results in two versions of PCR2 (PRC2.1 and PRC2.2), and some PcG proteins like PCL1/2/3 play crucial roles in their formation [15]. Among the subunits of PRC1/2, EZH2 and RING1 are the most important catalytic subunits function in PRC2 and PRC1, respectively. In addition, BMI1 is also an indispensable cofactor for the H2A ubiquitin ligase activity of RING1 [16].

Recently, the alteration of PcG proteins has been linked to tumorigenesis and metastasis [17–19]. In mechanism, aberrant expression of PcG protein-coding genes, driven by their methylation and/or copy number variation (CNV), could affect the function of the PcG complex and provide epigenetic prerequisite for malignant transformation [20–22]. However, few studies take all of these aspects (mRNA expression, DNA methylation and gene copy number variation) into consideration when referring to the influence of PcG proteins on the initiation and progression of cancer. All these intrigue us to analyze the whole PcG proteins as an integrity and study downstream alteration in gene expression and global phenotype.

Here, we systemically incorporated DNA methylation, mRNA expression, and CNV profiling of PcG protein-coding genes (PcG genes) from the HCC database. Based on the multi-omics analysis, we concluded different subtypes with distinct phenotypes. Meanwhile, we analyzed the relation between those subtypes and patients’ clinical features and outcomes. Experimentally, we explored the key molecule whose aberrant expression results in different HCC subtypes. In summary, we developed a novel classification based on PcG genes which have potential prognostic and therapeutic value for HCC patients.

## Materials and methods

### Patient tissues obtaining

A total of 56 HCC tissues were obtained from patients who received liver biopsy partial hepatectomy or liver transplantation at First Affiliated Hospital of Xi’an Jiaotong University, and pathological data were obtained from the pathology department. Follow-up data within 5 years were collected, and survival analysis was conducted through Kaplan–Meier analysis with a log-rank test. Ethical approval was obtained from the ethics committee of First Affiliated Hospital, and informed consent was obtained from each patient.

### Data collection and analysis, establishment of subtype model and other bioinformatics methods.

Gene expression data, DNA methylation data, and copy number data were obtained from UCSC Xena public database in a standard format. After patient code and gene ID matching, 372 patients with mRNA expression data of 447 PcG-related genes, DNA methylation data of 1109 PcG-related genes, and copy number data of 429 PcG-related genes were incorporated into subtype analysis. CancerSubtypes (version 1.8.0) were used to establish multi-omics HCC subtypes. Three clustering analyses (consensus clustering, CC, similarity network fusion, SNF, and non-negative matrix factorization, NFM) were performed, and the optimal clustering number was chosen according to the average silhouette value. Gene set variation analysis (GSVA) and oncoplot analysis were performed by GSVA (version 1.42.0) and maftools (version 2.10.05) on R software (version 3.5.1).

### Immunohistochemistry assay (IHC)

HCC tissues from patients and subcutaneous tumors from nude mice were fixed in 4% paraformaldehyde for at least 48 h followed by dehydration and embedding in paraffin. Tissues were cut into sections for immunohistochemistry analysis. After deparaffinization with xylene, sections were subjected to antigen repair twice for 15 min at 100 °C with an interval cooling at room temperature for 5 min in citrate buffer (pH = 6.0). Tissue sections were blocked by 10% goat serum (Sigma-Aldrich) for 30 min and incubated with appropriately diluted primary antibody overnight at 4 °C (Additional file 1: Table S1). After being washed with PBS 3 times, tissue sections were incubated with the avidin–biotin complex for 20 min and were developed with diaminobenzidine. Finally, the tissue sections were counterstained with hematoxylin and mounted for observation. The proportion of high-positive (HP), positive (P), low-positive (LP), and negative cells were measured by the IHC profiler in Image J software (version 1.8.0). IHC scores were calculated by “IHC score = 300 × HP proportion + 200 × P proportion + 100 × LP proportion”.

### Cell culture and treatment

Human HCC cell lines (MHCC-97L and Huh7) were purchased from the Culture Collection of the Chinese Academy of Sciences (Shanghai, China). Cells were maintained in DMEM medium supplemented with 10% fetal bovine serum, 100U/mL penicillin, and 100 μg/mL streptomycin to prevent contamination. Cells were cultured at 37 °C in a humidified atmosphere with 5% CO2. Oxaliplatin (HY-17371, MedChemExpress, 5 μg/ml), GSK126 (HY-19817, MedChemExpress, 2 μM), and PRT4165 (HY-19817, MedChemExpress, 10 μM) were dissolved in PBS or DMEM and diluted to an appropriate concentration when used.

For glucose deprivation treatment, HCC cells were seeded into 96-well plates and were maintained in a standard medium. After cell adhesion, the standard medium was replaced by a glucose-free culture medium with the palmitic acid supplement or not (10 μM). After 24 h of culture, cell viability was measured by CCK-8 assay.

### RNA isolation, primer design and quantitative reverse transcription–polymerase chain reaction (qRT-PCR)

Total RNA from cells and patient tissues was extracted using Trizol (Invitrogen). Reverse transcription was performed according to the PrimeScript RT Master Mix protocol (TaKaRa, Mountain View, USA). Primers were designed using Primer-BLAST (NCBI) and were synthesized by GenePharma (Shang Hai, China). The primer sequences are shown in Additional file 1: Table S1. TB Green Premix Ex Taq (TaKaRa) was used to perform qPCR. The β-actin (ACTB) gene was used as the internal control.

### Western blot

Total protein was extracted from tissues and cells by radio immunoprecipitation assay (RIPA) lysis buffer (Beyotime). After concentration determination by BCA Protein Assay Kit (Beyotime), the protein was mixed with loading buffer (Beyotime) and heated to 100 °C for 10 min for denaturation. Protein (5 μg for the internal control and 20 μg for targets) was loaded into polyacrylamide gels and separated by electrophoresis. Protein was then transferred to polyvinylidene difluoride membranes. Membranes were incubated overnight at 4 °C with primary antibodies diluted in 10% milk (Additional file 1: Table S1). After being washed three times with PBS with 1% Tween (PBST), membranes were incubated with secondary antibodies for 30 min at room temperature. The membranes were washed 3 times with PBST before chemiluminescence.

### Drug sensitivity measurement

Drug sensitivity measurement was based on the cell viability of HCC cells treated with oxaliplatin with a concentration of 0, 0.1, 0.5, 1.0, 5.0, 10.0, and 20.0 μg/mL, which was measured by CCK-8 assay. Cells were seeded into the 96-well plate with a density of 5000/well one day before pre-treatment of GSK126 or/and PRT4165. After 24 h of pre-treatment, HCC cells were washed with PBS 3 times. A total of 10 μL CCK-8 and 90 μL serum-free DMEM were added to each well, and the microplate was incubated at 37 °C for 1 h. The optical density at 450 nm was read with a 96-well multi-scanner plate reader (Biotech Instruments). Cell viability was calculated by the ratio of the optical density subtraction of treated and blank wells to the optical density subtraction of untreated and blank wells.

### Gene overexpression and interference

Knockdown or overexpression of CBX2 in Huh7 and MHCC-97L cell lines was achieved by siRNA targeting CBX2 mRNA or overexpression plasmid. SiRNA and plasmid were designed and synthesized by GenePharma (Shanghai, China) according to a previous study and were delivered into cells by Lipofectamine 2000 (Additional file 1: Table S1) [23].

### Immunofluorescence assay and lipid visualization

For lipid visualization, HCC cells were seeded in confocal dishes, after stable adherence cells were treated by GSK126 or PRT4165 for 24 h. Then, cells were stained by BODIPY (HY-125746, MedChemExpress) with concentration of 1 μM at 37 °C for 20 min and Hoechst 33,342 (Beyotime) at 37 °C for 5 min before observation by microscope. Particle analysis was performed by Images J software (version 1.8.0).

### Cell apoptosis measurement

Cell apoptosis was measured by flow cytometry analysis. HCC cells were seeded into 6-well plates and pretreated with GSK126, PRT4165, or together before being treated with 5 μg/mL oxaliplatin. After 24 h of oxaliplatin treatment, HCC cells were harvested with PBS three times. Then, HCC cells were stained with Annexin V-PE and 7-aminoactinomycin D (7AAD, BD) according to the manufacturer protocol and analyzed for fluorescenceusing a flow cytometer (FACSCalibur, BD). The proportion of Annexin V-PE-positive cells was used to indicate the cell apoptosis rate.

### Fatty acid oxidation measurement

Fatty acid oxidation measurement assay was based on the Fatty Acid Oxidation Assay (ab217602, Abcam, USA) and Extracellular Oxygen Consumption Assay (ab197243, Abcam, USA) according to manufacture’s protocol. HCC cells were seeded into 96-well plates and were maintained in standard medium overnight. After adherence, cells were pre-treated by GSK126 or PRT4165 and were maintained in glucose-deprivation (glucose-free) medium for 24 h. Then, cells were gently washed with fatty acid-free medium. Pre-warmed fatty acid measurement medium and extracellular O2 consumption reagent were added and wells were sealed by oil. The optical signal was measure after 60 and 120 min by multi-scanner plate reader. Fatty acid oxidation effect was calculated by signal ratio of negative control group with inhibitor-treated group subtracted by background of blank well.

### In vivo studies

Athymic nude mice were purchased from the Laboratory of Animal Research Center of Xi’an Jiaotong University. The xenograft tumor model was established in mice by subcutaneous inoculation with 1 × 10^6^ Huh7 cells. The oxaliplatin administration was set on the 20th day after inoculation of HCC cells (5 mg/kg). On the 30th day, the mice were killed and the tumors were harvested, weighed, and photographed. The protocol of animal experiments was approved by the Institutional of Animal Care and Use Committee at Xi’an Jiaotong University. The tumor volume was calculated by the formula: V = Length × Width^2^ × 0.5.

### Statistical analysis

All analyses were performed based on GraphPad Prism (Version 8.0). Data represent mean ± SD from 3 repeated experiments. The Student’s t-test was used for comparing quantitative data. One-way ANOVA was used for testing differences across quantitative data of multiple groups. The ranked data were analyzed with the Chi-square test. Pearson correlation analyses were performed to analyze the correlation of gene expression.

## Results

### Identification of two epigenetic subtypes of HCC based on PcG-related genes

**Fig. 1:**
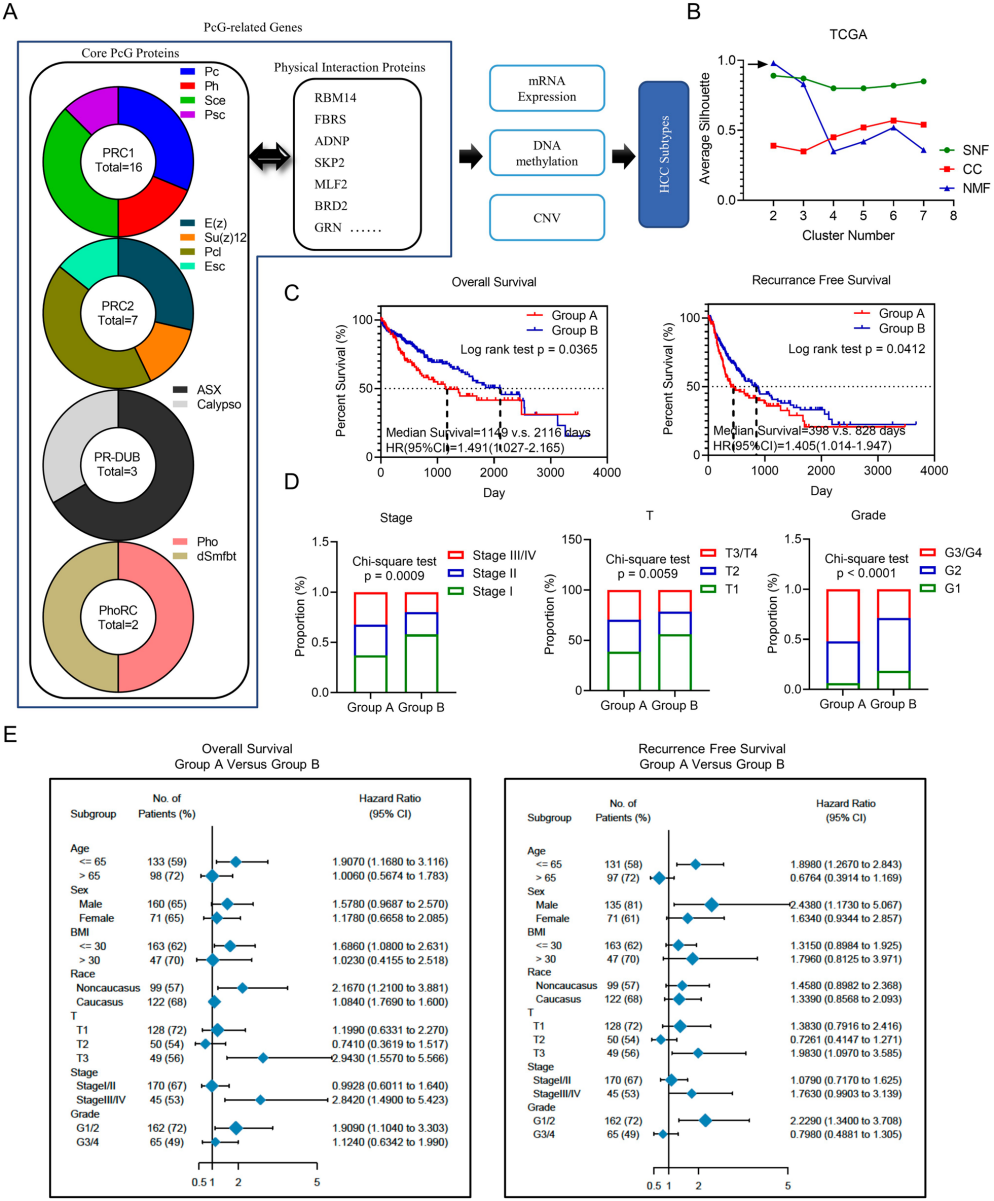
Identification of two epigenetic subtypes of HCC based on PcG-related genes. **A** The flow diagram of establishment of epigenetic classification of HCC based on bioinformatics analysis. **B** Average silhouette values of different clustering method and cluster number. The black arrows indicate optimized clustering strategy. NFM: non-negative matrix factorization; CC: consensus clustering; SNF: similarity network fusion. **C** Survival analysis of HCC patients in two epigenetic subtypes. **D** Comparison of clinicopathologic feature of HCC patients in different epigenetic subgroups. **E** Stratification analysis of HCC patients in different epigenetic subgroups according to their clinical data

Accumulating evidence suggests that besides core subunits like EZH2 and BMI1, numerous accessary proteins participate in formation of PcG complexes, significantly affecting PcG complexes` function [24, 25]. Therefore, in this study, aiming to analyze the subtype of HCC from the perspective of epigenetic regulation of the PcG complex in a more comprehensive picture, we included all the core and accessary proteins of four PcG complexes. We firstly summarized a total of 448 genes (PcG-related genes) which were reported to associate with core PcG proteins [26] (Fig. 1A). Combining the RNA sequencing, DNA methylation and CNV information of these 448 PcG-related genes, we then performed multi-omics analysis. We used three well-established algorithms, non-negative matrix factorization (NFM), consensus clustering (CC) and similarity network fusion (SNF), encapsulated in CancerSubtypes package of R software. We chose the appropriate cluster number with highest average silhouette to develop HCC classification and identified two novel subtypes of HCC (Groups A and B) (Fig. 1B). The two subtypes had distinct clinical outcomes and patients in Group A had a worse prognosis (Fig. 1C). Moreover, a higher proportion of advanced stage and malignant phenotype is shown in Group A (Fig. 1D). Subgroup analysis showed that the prognostic differences of two subtypes were more significant in male, younger, non-Caucasian patients with advanced HCC (Fig. 1E). Above results indicated epigenetic subtype based on PcG-related gene could predict the survival of HCC patients and reflected the biological characteristic of HCC cells.

In addition, we estimated the profile of tumor immune microenvironment in two subtypes. We found that HCC tissues of Group A exhibited a lower infiltration of NKT cells, neutrophil and endothelial cells, and a higher infiltration of B and Treg cells, consistent with their higher stroma and microenvironment scores (Additional file 2: Fig. S1). These results indicated Group A presented an immunosuppressive and non-inflammatory microenvironment. Meanwhile, we also analyzed mutation data in different subtypes and found that the hotspot mutations mainly enriched in Wnt/β-catenin pathway member genes in Group B, while enriched in genes associated with genome integrity like *TP53* in Group A (Additional file 3: Fig. S2A). Intriguing, we also found several HCC tissues in Group A exhibited extremely higher tumor mutation burden, which indicated epigenetic subtype could affect mutation. We also analyzed the DNA methylation, RNA sequencing and CNV profile of PcG genes between Group A and Group B. We found that CNV showed the most significant distinction between Group A and Group B. Group B had more disorder PcG genes CNV with higher amplification and deletion (Additional file 3: Fig. S2B).

### Two epigenetic subtypes had discrepant metabolic signatures

**Fig. 2:**
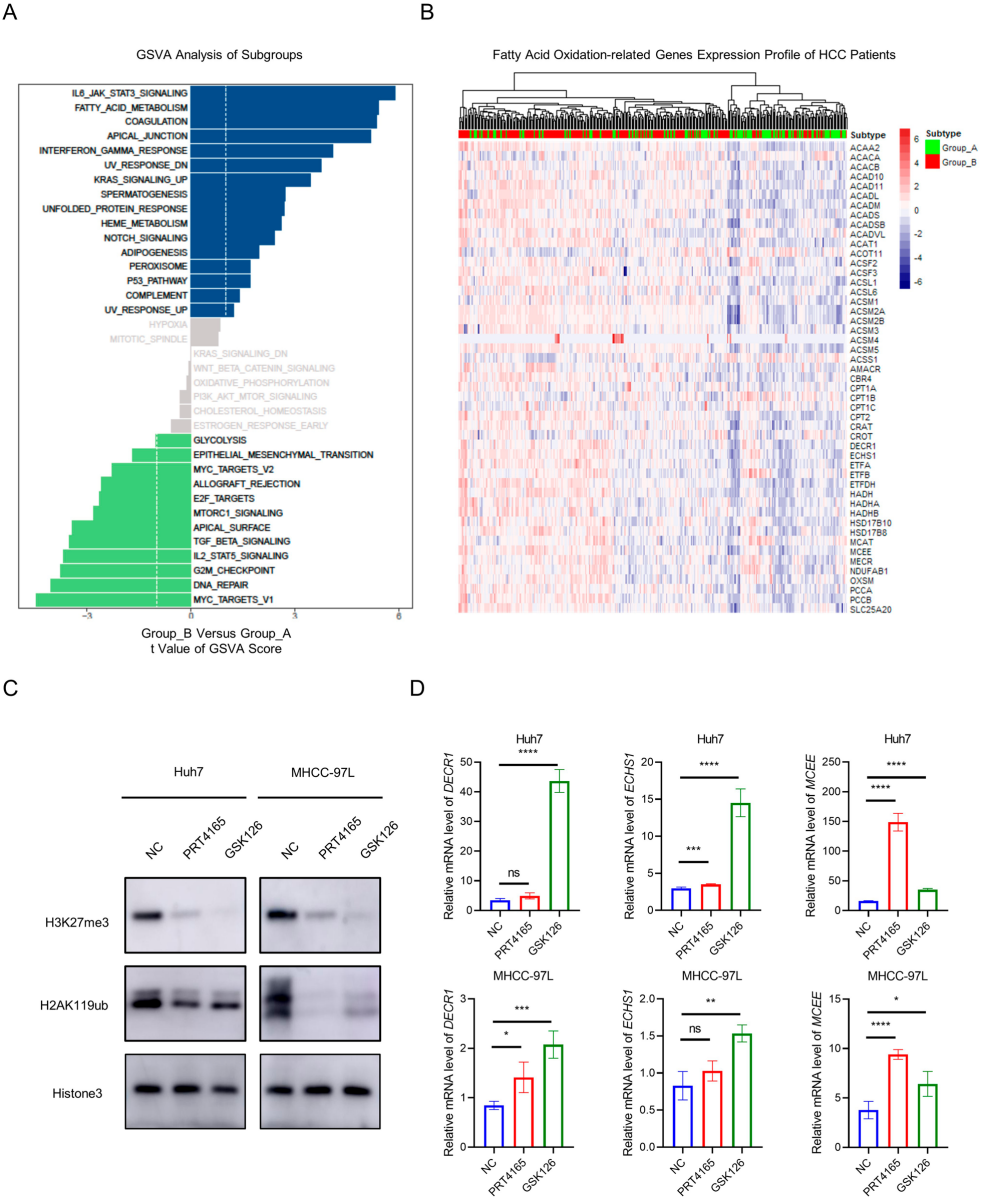
Two epigenetic subtypes had discrepant metabolic signatures. **A** Gene Set Variation Analysis (GSVA) of mRNA expression profile in HCC of different epigenetic subgroups. **B** Fatty acid oxidation-related genes expression profile of HCC in different epigenetic subgroups. **C** The downregulation of H3K27me3 and H2AK119ub protein level in HCC cells after GSK126 or PRT4165 treatment. **D** The downregulation of three fatty acid oxidation-related genes mRNA expression in HCC cells after GSK126 or PRT4165 treatment. β-actin (ACTB) was used as internal control. Ns, not significant; *p < 0.05; **p < 0.01; ***p < 0.005; ****p < 0.001

To further investigate the underlying molecular and biological discrepancy of two subgroups, we performed transcriptome sequencing analysis using the GSVA method. Multiple signaling pathways were involved in the varying clinical characteristics of two subgroups, including inflammatory, DNA damage and repair, radiosensitivity and especially cell metabolism pathways (Fig. 2A). Group A exhibited higher level of glycolysis activity, while lower fatty acid metabolism activity. We then detected the expression of fatty acid oxidation-related genes (FAORGs) in different subtypes. We found most of FAORGs were downregulated in Group A while overexpressed in Group B (Fig. 2B). Meanwhile, glycolysis-related genes exhibited inverse expression pattern in two groups (Additional file 4: Fig. S3A). Intriguingly, correlation analysis showed that the expression of most FAORGs was negatively associated with PRC1/2 subunits genes, which indicated that these genes were epigenetic suppressed in Group A HCC patients (Additional file 4: Fig. S3B). To validate this mechanism, we used PRT4165 and GSK126 to inhibit PRC1 and PRC2 activity, which decreased H3K27me3 and H2AK119ub deposition, respectively (Fig. 2C). We found that PRC1/2 inhibition restored FAORGs expression (e.g., *MCEE*, *ECSH1* and *DECR1*) (Fig. 2D). These results indicated two HCC subtypes had discrepant metabolic signatures, especially in lipid and glycolytic metabolism pathways, which were affected by the activity of PcG complex.

### Modulation of PcG activity affected metabolic signatures of HCC cells

**Fig. 3:**
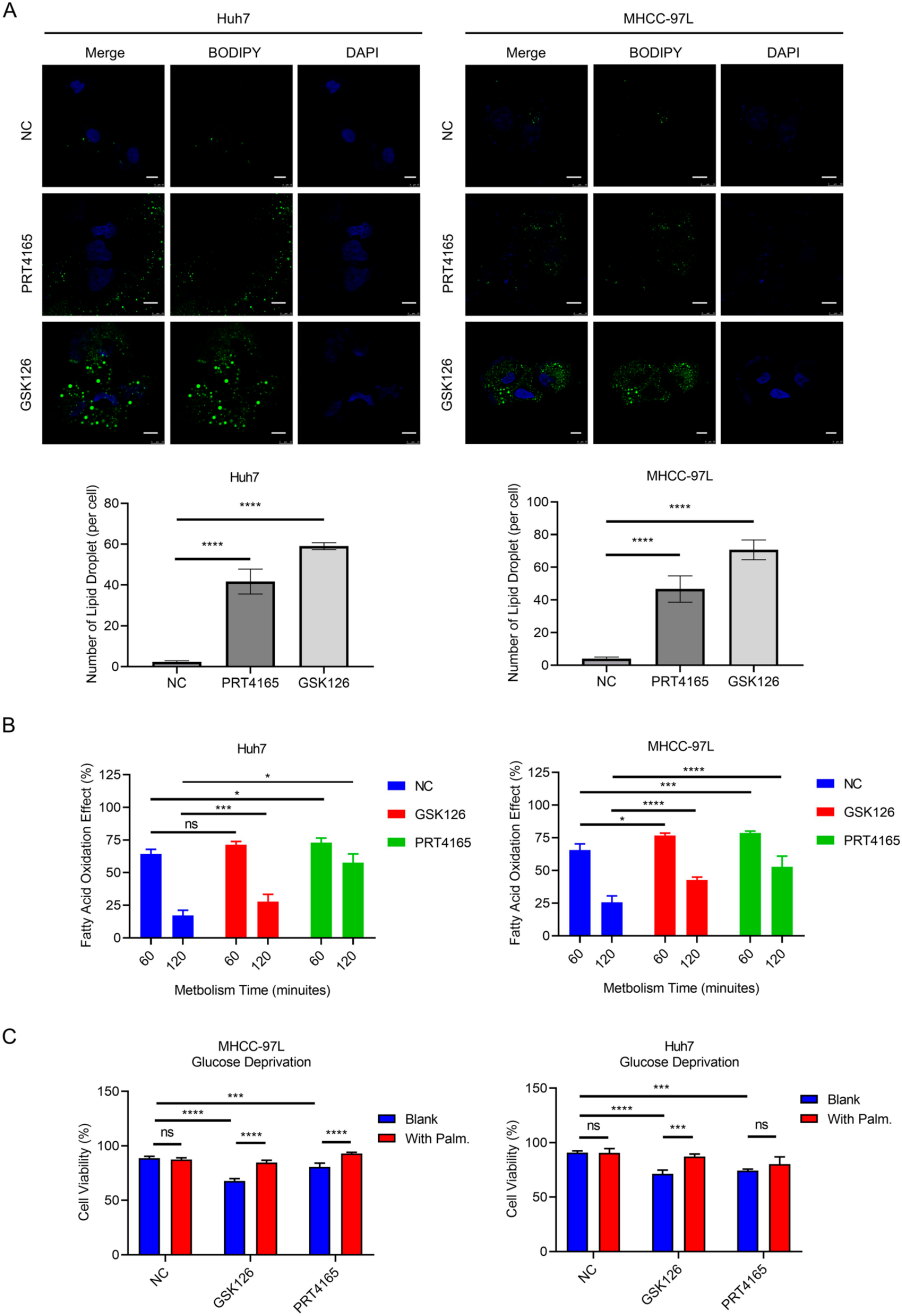
Modulation of PcG activity affected metabolic signatures of HCC cells. **A** The lipid fluorescence staining of HCC cells after GSK126 or PRT4165 treatment. The white arrow indicated 10 μm. Three independent assays were analyzed and average number of lipid droplet of each field was measured. ****p < 0.001. **B** The fatty acid oxidation effect of HCC cell after GSK126 or PRT4165 treatment. Three independent assays were performed, and data were collected at 60- and 120-min time points. Ns, not significant; *p < 0.05; ***p < 0.005; ****p < 0.001. **C** Cell viability of HCC cells after glucose deprivation with or without treatment of GSK126 or PRT4165 and after supplement of palmitic acid. Three independent assays were performed. Ns, not significant; *p < 0.05; ***p < 0.005; ****p < 0.001

To further validate the effect of the PcG activity on fatty acid metabolism, we detected the deposition of lipid droplets in HCC cells after modulating the activity of the PcG complex. We found that PRC1/2 inhibitors could remarkably promote lipid accumulation in HCC cells (Fig. 3A). Then, we measured the activity of fatty acid oxidation in HCC cells under PRC1/2 inhibitors treatment and found that both GSK126 and PRT4165 efficiently strengthened the lipid catabolism within 120 min (Fig. 3B). Meanwhile, we also investigated the influence of PRC1/2 inhibition on cell viability under energy stress. We found that glucose deprivation significantly inhibited cell viability under treatment of PRC1/2 inhibitors (Fig. 3C). In contrast, palmitic acid supplement restored the synthetic lethal effect of PRC1/2 inhibition and glucose shortage (Fig. 3C). Above results supported that modulation of PcG activity induced metabolic reprogramming of HCC cells, leading them addict to lipid metabolism but more fragile under glucose shortage, and consequently affecting tumor progression.

### Inhibition of PcG complex sensitizes HCC cells to chemotherapy

**Fig. 4:**
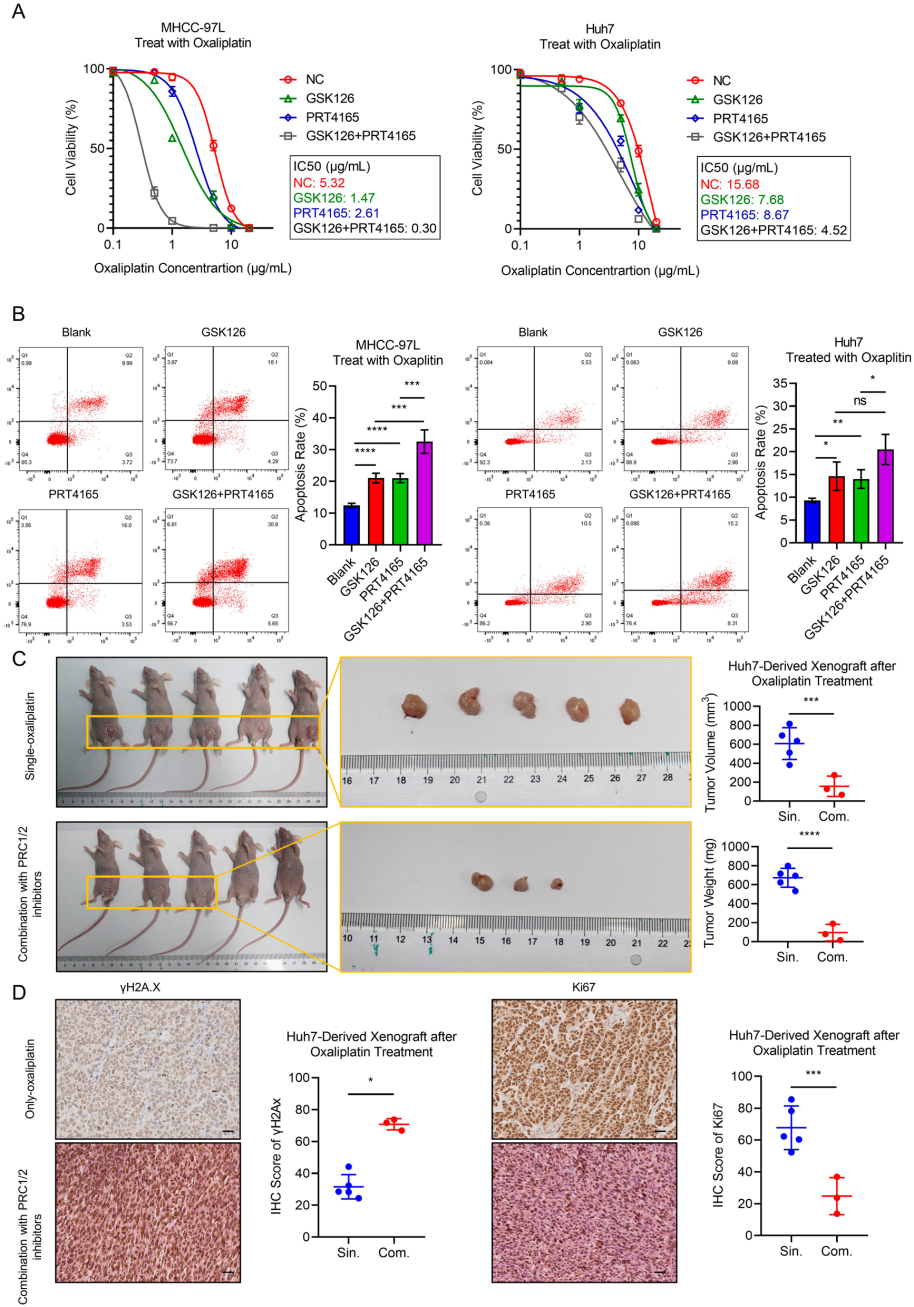
Inhibition of PcG complex sensitizes HCC cells to chemotherapy. **A** Cell viability of HCC cells under treatment of oxaliplatin together with GSK126 or PRT4165. The signal from three independent wells were collected and 50% inhibitory concentration (IC50B) was calculated by four-parameter model. **B** Cell apoptosis rate of HCC cell after oxaliplatin treatment (5 μg/ml) with GSK126 or PRT4165 measured by flow cytometry**.** Ns, not significant; *p < 0.05; **p < 0.01; ***p < 0.005; ****p < 0.001. **C** Huh7-cell derived subcutaneously tumor after treatment of oxaliplatin with or without GSK126 and PRT4165. ***p < 0.005; ****p < 0.001. **D** The IHC assay of γH2A.X and Ki67 subcutaneously tumor after low-dose oxaliplatin treatment with or without GSK126 and PRT4165. The black bar represents 100 μm, and *p < 0.05; ***p < 0.005

The GSVA results predicted that the patients in Group A who have overexpressed DNA repair-related genes had a higher sensitivity to chemotherapy. We then investigated the effect of PcG complex inhibition on platinum-based drugs. Here, we treated HCC cells with oxaliplatin, a widely used chemotherapeutic drug for HCC [27]. We found that treatment of PRT4165 and/or GSK126 sensitized HCC cells to oxaliplatin (Fig. 4A). In addition, we detected cell apoptosis after combination treatment of PRC1/2 inhibitors and oxaliplatin (5 μg/ml) and found PRC1/2 inhibitors enhanced cell sensitivity to oxaliplatin treatment and resulted in a higher proportion of cell apoptosis (Fig. 4B). Furthermore, we validated above findings in vivo. After a 10-day course of combination (oxaliplatin + PRC1/2 inhibitors) or single oxaliplatin treatment, we found the combination group had significantly smaller volumes and weights than the single oxaliplatin treatment group (Fig. 4C). Moreover, we compared the expression of γH2A.X (a biomarker for chemotherapy-induced DNA damage) and Ki67 (a biomarker for cell proliferation) between two treatment groups and found that combination treatment group had higher γH2A.X and lower Ki67 expression than single oxaliplatin treatment group (Fig. 4D). Taken together, our data showed that inhibition of PcG complexes enhanced HCC cell sensitivity to oxaliplatin.

### Overexpression of CBX2 contributed to aberrant epigenetic phenotype of HCC cells

**Fig. 5:**
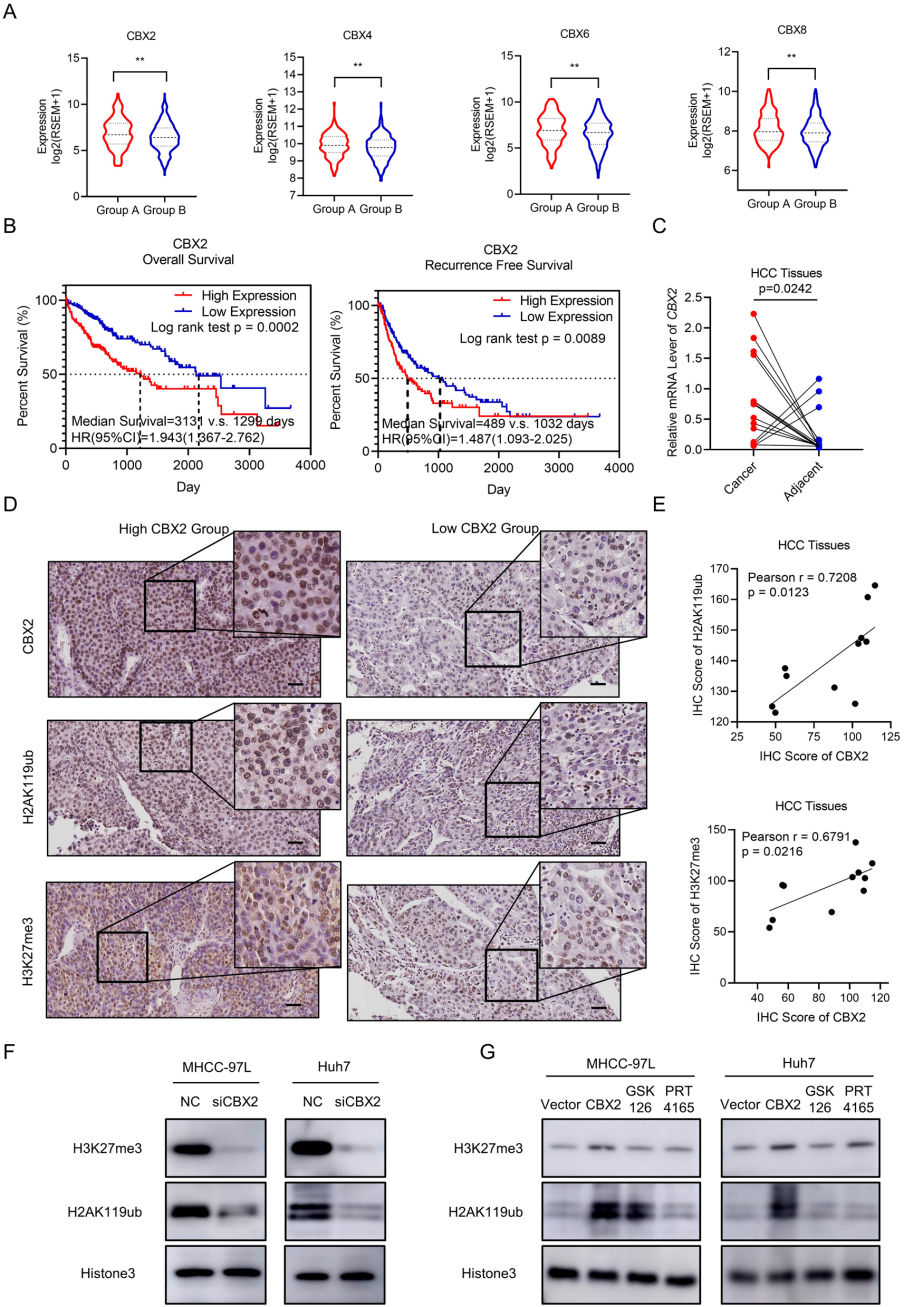
Overexpression of CBX2 contributes to aberrant epigenetic landscape of HCC cells. **A** The mRNA expression of PcG genes of HCC patients in different epigenetic subgroups. **p < 0.01; ****p < 0.001. **B** The Kaplan–Meier curve based on survival analysis of HCC patients expressing high or low level of CBX2. **C** The mRNA level of CBX2 in tumor and adjacent tissue of HCC patients. The relative mRNA level was normalized by β-actin (ACTB). **D** The IHC assay of CBX2, H3K27me1 and H2AK119ub in high and low CBX2 expression HCC groups. The black bars represent 200 μm. **E** The correlation analysis of CBX2 expression with H3K27me3 or H2AK119ub expression. The Pearson coefficients were calculated. **F** The immunoblot of H3K27me1 and H2AK119ub in CBX2 knockdown HCC cells. **G** The immunoblot of H3K27me3 and H2AK119ub in CBX2 overexpression HCC cells

We then investigated the molecular mechanism of the epigenetic subtype. We compared the expression of the PcG gene in two HCC subtypes and found that CBX family coding genes (*CBX2*, *CBX4*, *CBX6,* and *CBX8*) and *EZH2* were upregulated in Group A (Fig. 5A). Through survival analysis, we found that CBX2 expression is a poor prognostic factor for HCC patients (Fig. 5B). We also identified upregulation of CBX2 in cancer tissues and its association with HCC stage (Fig. 5C and Additional file 5: Fig. S4). Previous research proved that CBX2 could mediate the cross-talk between PRC1 and PRC2 and facilitate H3K27me3, and H2AK119ub deposition [28]. Therefore, we reasoned that CBX2 overexpression might be responsible for excessive epigenetic modulation and result in an aberrant epigenetic phenotype. We measured the expression of CBX2, H3K27me3 and H2AK119ub in HCC tissues using immunohistochemistry stains (Fig. 5D). We found both H3K27me3 and H2AK119ub were significantly positively correlated with CBX2 expression (Fig. 5E). In HCC cells, both of H3K27me3 and H2AK119ub were shown to be upregulated after overexpressing CBX2, and both of them were then downregulated after CBX2 inhibition (Fig. 5F, G and Additional file 6: S5). In summary, we proved that CBX2 was a crucial contributor to aberrant epigenetic phenotypes in HCC cells and was associated with HCC epigenetic classification.

## Discussion

It is well established that aberrant expression of core PcG subunits contributes to tumor initiation and progression. In HCC, overexpression of EZH2 represses miR-622 through H3K27me3 deposition and results in CXCR4 upregulation and unfavorable prognosis, while BMI1 enhances TGFβ2/SMAD pathway and facilitates tumor cell proliferation and cell cycle progression [29, 30]. In recent years, accumulating evidence suggested that other non-core accessory proteins alternatively constituting the PcG complex can also facilitate the pro-tumor process. For example, euchromatic histone lysine methyltransferase 2 (EHMT2), an alternative subunit of PRC1.6., has been recently reported to facilitate HCC progression and aggressive features through epigenetic silencing of tumor suppressor genes [31]. In addition, although PR-DUB and PhoRC are scarcely reported in the field of HCC study, based on their close relationship with PRC1/2, we supposed that they are also functional in the epigenetic classification of HCC [32]. According to our classification, patient in two subgroups has a significantly distinct prognosis and exhibit different biological features, which is related to aberrant PcG complex activity and epigenetic landscape.

Abnormal histone modifications, especially methylation and ubiquitination, are associated with poor prognosis of cancer patients and decide biological phenotypes of tumor tissue [33–35]. It has been reported that histone methylation is associated with various malignant features like vascular invasion, large tumor size, multiplicity as well as relapse in HCC [36]. A recent review also comprehensively summarized that the HCC-promoting function of histone ubiquitination was related to transcription repression and could be also resulted from the upregulation of the ubiquitin-specific peptidase family, which we also included in our present work [37]. Aberrant expression of PcG proteins like BMI1, CBX8, and EZH2 is mainly responsible for the deposition and maintenance of pro-tumor histone modification in HCC [30, 38]. In our study, we identified two subtypes of HCC by the analysis of the expression pattern of PcG-related genes, and CBX8 and EZH2 are upregulated in group A. Thus, we referred that Group A HCC had elevated histone methylation and ubiquitination. According to survival analysis, patients in group A have a worse prognosis, especially in young and stage III/IV subpopulation. These results suggest that the poor prognosis of Group A patients is related to pro-tumor histone modification, which is consistent with previous studies.

According to our informatics analysis, HCC cells in two subgroups have distinct characteristics in many aspects, especially cell metabolism. We found that patients in Group A have lower expression of the fatty acid metabolism gene, and the downregulation of these genes can be abolished by the PRC1/2 inhibitors. Consistently, the capacity of fatty oxidation and accumulation of HCC cells were also promoted after PRC1/2 inhibitors treatment. In addition, our results showed the increased dependence on lipids and sensitivity to glucose deprivation of PRC1/2-inhibited HCC cells. In recent years, many studies have suggested that metabolic homeostasis is crucial for HCC cells in a setting of reduced nutrient availability, which can be affected by epigenetic regulation [39–41]. Recently, Li et al. [39] proved that the deficiency of SET domain-containing 2 (SETD2), a histone methyltransferase, could cause downregulation of H3K36me3 and cholesterol efflux genes, which led to lipid accumulation and HCC development. In our study, we focused on H3K27me3 and H2AK119ub, which are regulated by PcG complexes and found these histone modifications have a broad influence on the aspect of fatty acid metabolism. Intriguingly, the downregulation of H3K27me3 and H2AK119ub alters HCC cells’ preference of energy source from glucose toward fatty acid and makes them more fragile under the shortage of glucose, which impairs their capacity for metabolic adaption. However, whether epigenetic regulation of metabolic adaption could affect the initiation and progression of HCC needs further investigation.

The drugs targeting mutant PcG proteins such as EZH2 have been extensively investigated, and several of them have been applied to preclinical research of hematological malignancy treatment [42, 43]. However, in solid tumors, aberrant function and excessive enzymatic activity of PcG proteins are usually derived from their overexpression instead of their mutation, where the efficiency of PcG targeting drugs is limited. To solve this problem, PcG protein-targeting drugs can be combined with immune therapy, conventional chemotherapy, or other targeting therapy [44, 45]. Here, we combined oxaliplatin, a widely used chemotherapeutic drug for gastrointestinal malignancy treatment, with PRC1/2 inhibitors respectively, or synergistically. Our data showed the efficiency of combined therapy to induce HCC cell apoptosis in vitro and inhibit tumor progression in vivo. In addition, our study proved that synergistic inhibition of PRC1 and PRC2 can bring higher sensitivity to oxaliplatin, and similar therapeutic strategies have recently been reported in glioblastomas treatment where the investigators successfully used molecular inhibitors of BMI1 and EZH2 to control proneural and mesenchymal tumors under the limits of detection [46]. Targeting PcG proteins dissolves the pro-tumor histone modification and enhances the sensitivity of cancer cells to other treatments. Recently, Wang et al. [47] made full use of DNA-encoded libraries (DELs) and discovered a novel CBX2-targeting inhibitor, SW2_152F, which could block the N-terminal chromodomain (ChD) and inhibit the binding of CBX2 and H3K27me3. Importantly, SW2_152F had high specificity to CBX2 compared to other CBX proteins and could reduce the H3K27me3 deposition on specific gene promoters. Additionally, SW2_152F has been proven to inhibit the growth of triple-negative breast cancer cells and the neuroendocrine differentiation of prostate cancer cells, which indicated that CBX2 inhibitor could be a pan-cancer therapeutic agent [47, 48]. Further research was needed to investigate the specific function of CBX2 in different types of human cancers by SW2_152F or other CBX2 inhibitors.

In our study, we proved that overexpression of CBX2, the epigenetic reader, contributes to overexpression of histone methylation and ubiquitination by promoting the activity of PRC1/2 in HCC cells. As a “bridge” of PRC1 and PRC2 intercommunication, CBX2 recognizes the H3K27me3 marker and recruits other subunits of PRC1 facilitating histone modification of H2AK119ub, which can be recognized by PRC2, which induces chromatin compaction for deep silence of genes [49, 50]. A previous study has reported the extranuclear function of CBX2 to activate the YAP pathway in HCC progression [23]. Here, our result showed the intranuclear function of CBX2 to promote the H3K27me3 and H2AK119ub deposition. A recent review summarized the therapeutic potential of targeting CBX2 and its advantages beyond directly targeting EZH2 [28]. Previous review also emphasized the importance of therapeutic strategy with dual- or multi- inhibitory activities on different epigenetic machines [37]. Consistently, our study provided evidence that knockdown of CBX2 could simultaneously alleviate two pro-cancer histone modification (H3K27me3 and H2AK119ub), and this therapeutic strategy may especially benefit HCC patients who bear an aberrant epigenetic landscape like subtype Group A patients.

## Conclusions

In conclusion, our study identified a novel classification strategy of HCC patient according to PcG-related gene expression and epigenetic landscape, which had significant prognostic value. Our experiment data showed the correlation between this classification system and biological features of HCC like cancer cell metabolism and chemotherapeutic sensitivity. We further found CBX2 overexpression in one subtype and proved that CBX2 could modulate epigenetic modification of histone in HCC. We proved that targeting CBX2 could be a feasible strategy to against aberrant PcG complex activity to inhibit HCC progression.

## Supplementary Information

**Additional file 1**.**Table**  **S1** The information of antibody and sequence of siRNA and primer for quantitative PCR assay.

**Additional file 2**. **Fig**. **S1** The tumor microenvironment analysis and immune cell infiltration analysis of two epigenetic subtypes based on mRNA expression profile.

**Additional file 3**. **Fig.** **S2** A. Gene mutation analysis of HCC patients of two epigenetic subgroups. B. CNV, DNA methylation and RNA expression of PcG genes in two HCC subgroups, the black boxes show the hot region with significant difference between two groups.

**Additional file 4**. **Fig.** **S3** Metabolism related gene expression profile of two HCC subtypes and its correlation with PRC1/2 subunits coding gene expression. A. Glycolysis related genes expression profile in HCC of different epigenetic subgroups. B. Expression correlation analysis of PRC1/2 genes with fatty acid oxidation-related genes and glycolysis related genes.

**Additional file 5**. **Fig.** **S4** The CBX2 is upregulated in HCC tissues. A. The immunohistochemistry assay of different stage HCC tissues. N=13 for Stage I, N=13 for Stage II, N=16 for Stage III and N=14 for Stage IV. The black bar represents 100μm. B. The representative immunoblots of CBX2 in tumor (T1~T4) and adjacent (A1~A4) tissue of HCC patients. The semiquantitative analysis were performed by integrated optical density measurement with Image J software.

**Additional file 6**. **Fig.** **S5** The CBX2 protein expression after knockdown and overexpression in HCC cells.

## Data Availability

The datasets analyzed for the current study are available from the corresponding author on reasonable request.

## Abbreviations

HCC: Hepatocellular carcinoma
PRC1/2: Polycomb-repressive complex ½
H3K27me3: Tri-methyl-histone H3 (Lys27)
H2AK119ub: Ubiquityl-histone H2A (Lys119)
PR-DUB: Polycomb-repressive deubiquitylase
PhoRC: Pho-repressive complex
CNV: Copy number variation
siRNA: Small interfering RNA
NFM: Non-negative matrix factorization
CC: Consensus clustering
SNF: Similarity network fusion
GSVA: Gene set variation analysis
qRT-PCR: Quantitative reverse transcription–polymerase chain reaction
FAORGs: Fatty acid oxidation-related genes
IHC: Immunohistochemistry assay
