# Supplementary material for: Identification of hepatocellular carcinoma subtypes based on PcG-related genes and biological relevance with cancer cells
Source: Clin Epigenetics. 2022 Dec 24;14:184. doi: 10.1186/s13148-022-01393-6 (PMC9790136; doi:10.1186/s13148-022-01393-6)
Supplement: Supplementary file 1 — Additional file 1.Table S1 The information of antibody and sequence of siRNA and primer for quantitative PCR assay. [file 13148_2022_1393_MOESM1_ESM.docx]

**Table S1**

The information of antibody and sequence of siRNA and primer for quantitative PCR assay.

| **Antibody** | | |
| --- | --- | --- |
|  | Dilution | Cat. Number |
| CBX2 | 1:1000 for western blot; 1:100 for IHC | Proteintech; 15579-1-AP |
| H3K27me3 | 1:1000 for western blot; 1:100 for IHC | Cell Signaling; #9733 |
| H2AK119ub | 1:2000 for western blot; 1:1500 for IHC | Cell Signaling; #8240 |
| Histone3 | 1:2000 for western blot; 1:1500 for IHC | Proteintech; 17168-1-AP |
| γH2A.X | 1:5000 for western blot; 1:250 for IHC | Abcam; ab81299 |
| Ki67 | 1:10000 for IHC | Proteintech; 27309-1-AP |
| **Small Interfering RNA** | | |
|  | Forward (5'->3') | Reverse (5'->3') |
| siNC | UUCUCCGAACGUGUCACGUTT | ACGUGACACGUUCGGAGAATT |
| siCBX2 | CCAGCCUAAUGAAGGGCAUTT | AUGCCCUUCAUUAGGCUGGTT |
| **Primer** | | |
| *DECR1* | GAGGTACTGGCCTTGGTAAAG | AATTTGTTCTGCGGTAGCTTTC |
| *ECHS1* | GCACAGCCGGAGATCTTAAT | TCACCAGTGAGGACCATCT |
| *MCEE* | GCTCCCATTCCAACAGTAAGAG | CTGCTATGGCTACATGGTTGAG |
| *GAPDH* | CTCCTCCACCTTTGACGCTG | TCCTCTTGTGCTCTTGCTGG |
| *ACTB* | TCACCATGGATGATGATATCGC | ATAGGAATCCTTCTGACCCATGC |
