# Supplementary figures and images for: Identification of hepatocellular carcinoma subtypes based on PcG-related genes and biological relevance with cancer cells
Source: Clin Epigenetics. 2022 Dec 24;14:184. doi: 10.1186/s13148-022-01393-6 (PMC9790136; doi:10.1186/s13148-022-01393-6)

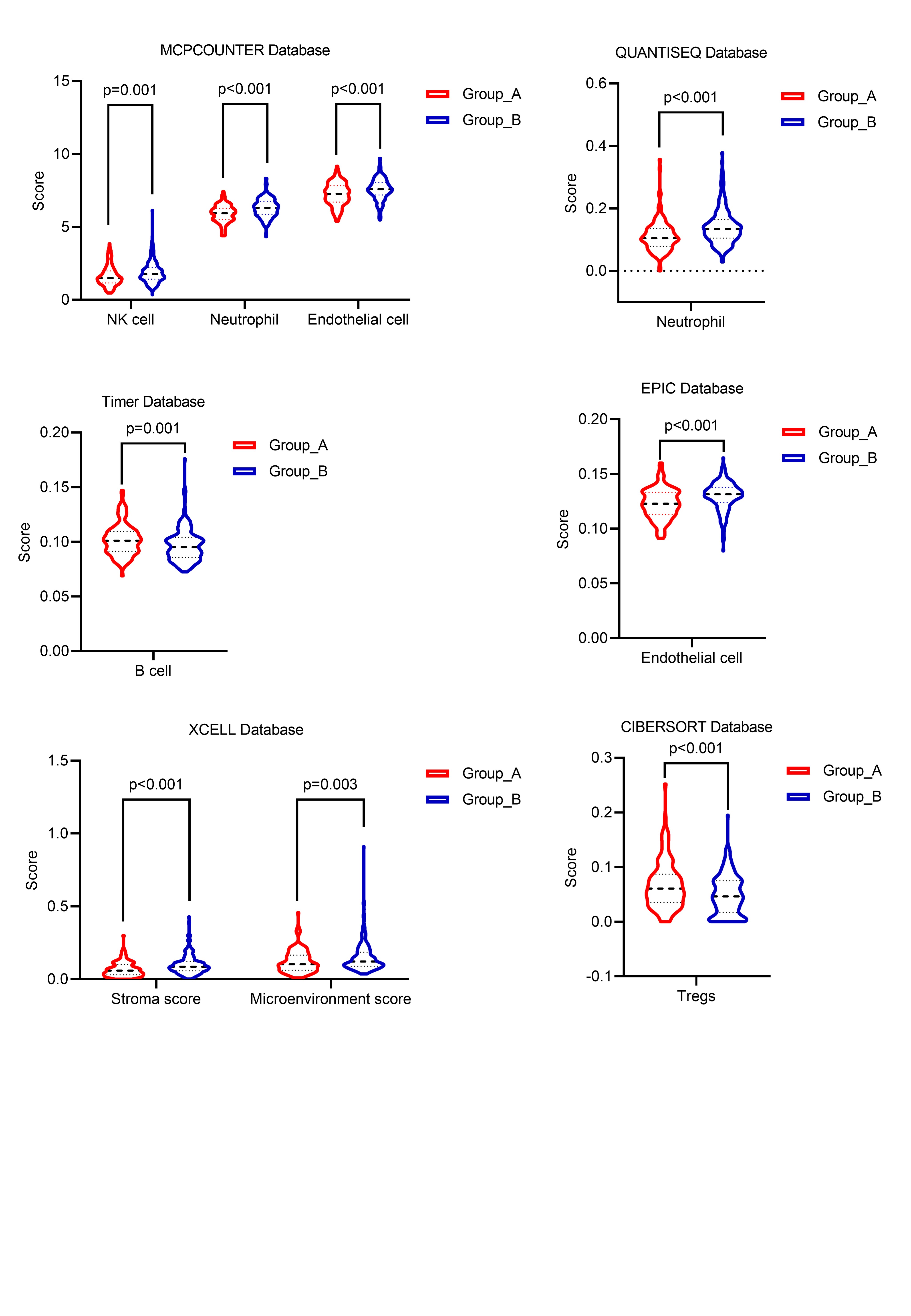

Supplement: Supplementary file 2 — Additional file 2. Fig. S1 The tumor microenvironment analysis and immune cell infiltration analysis of two epigenetic subtypes based on mRNA expression profile. [file 13148_2022_1393_MOESM2_ESM.jpg]

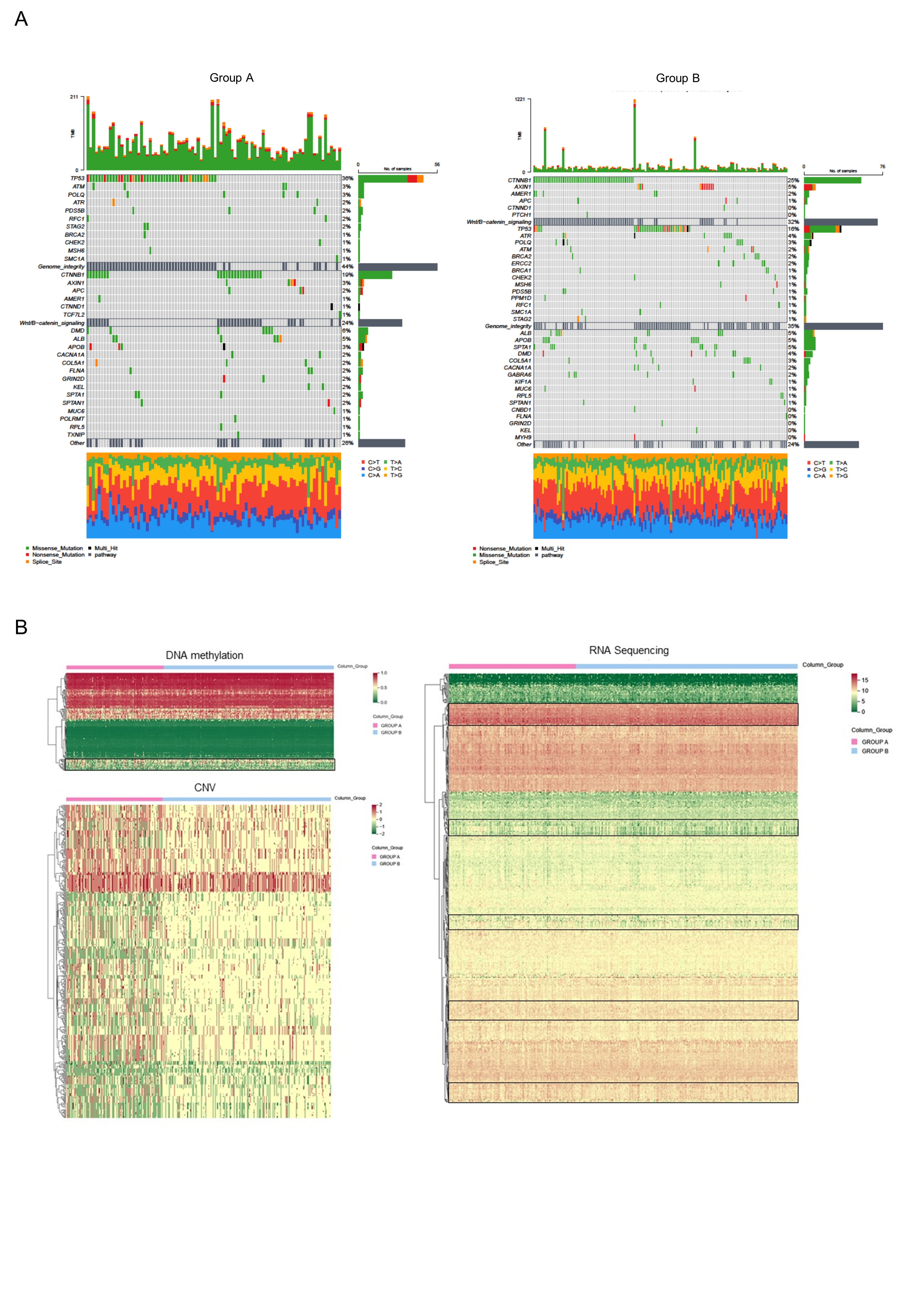

Supplement: Supplementary file 3 — Additional file 3. Fig. S2 A. Gene mutation analysis of HCC patients of two epigenetic subgroups. B. CNV, DNA methylation and RNA expression of PcG genes in two HCC subgroups, the black boxes show the hot region with significant difference between two groups. [file 13148_2022_1393_MOESM3_ESM.jpg]

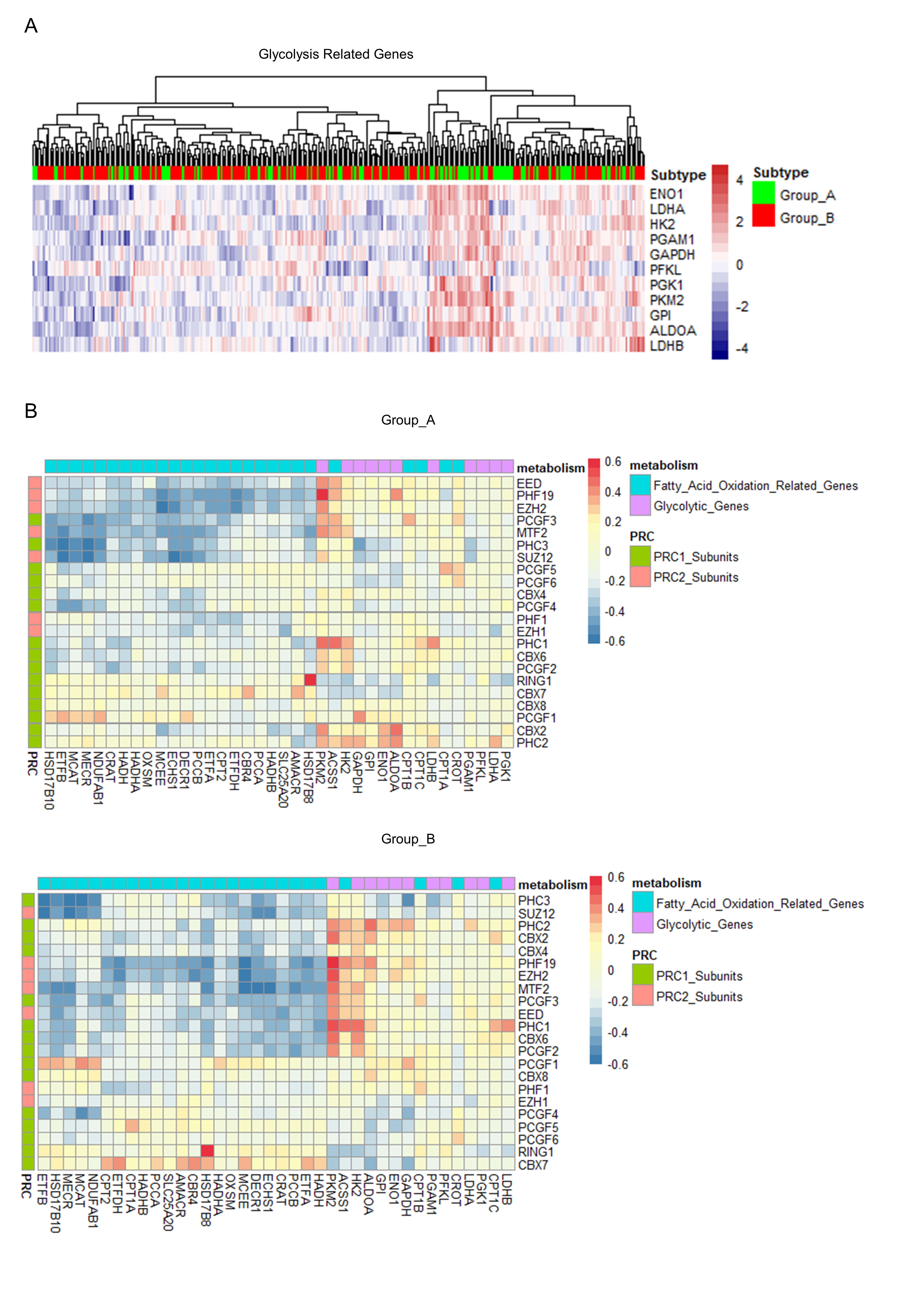

Supplement: Supplementary file 4 — Additional file 4. Fig. S3 Metabolism related gene expression profile of two HCC subtypes and its correlation with PRC1/2 subunits coding gene expression. A. Glycolysis related genes expression profile in HCC of different epigenetic subgroups. B. Expression correlation analysis of PRC1/2 genes with fatty acid oxidation-related genes and glycolysis related genes. [file 13148_2022_1393_MOESM4_ESM.jpg]

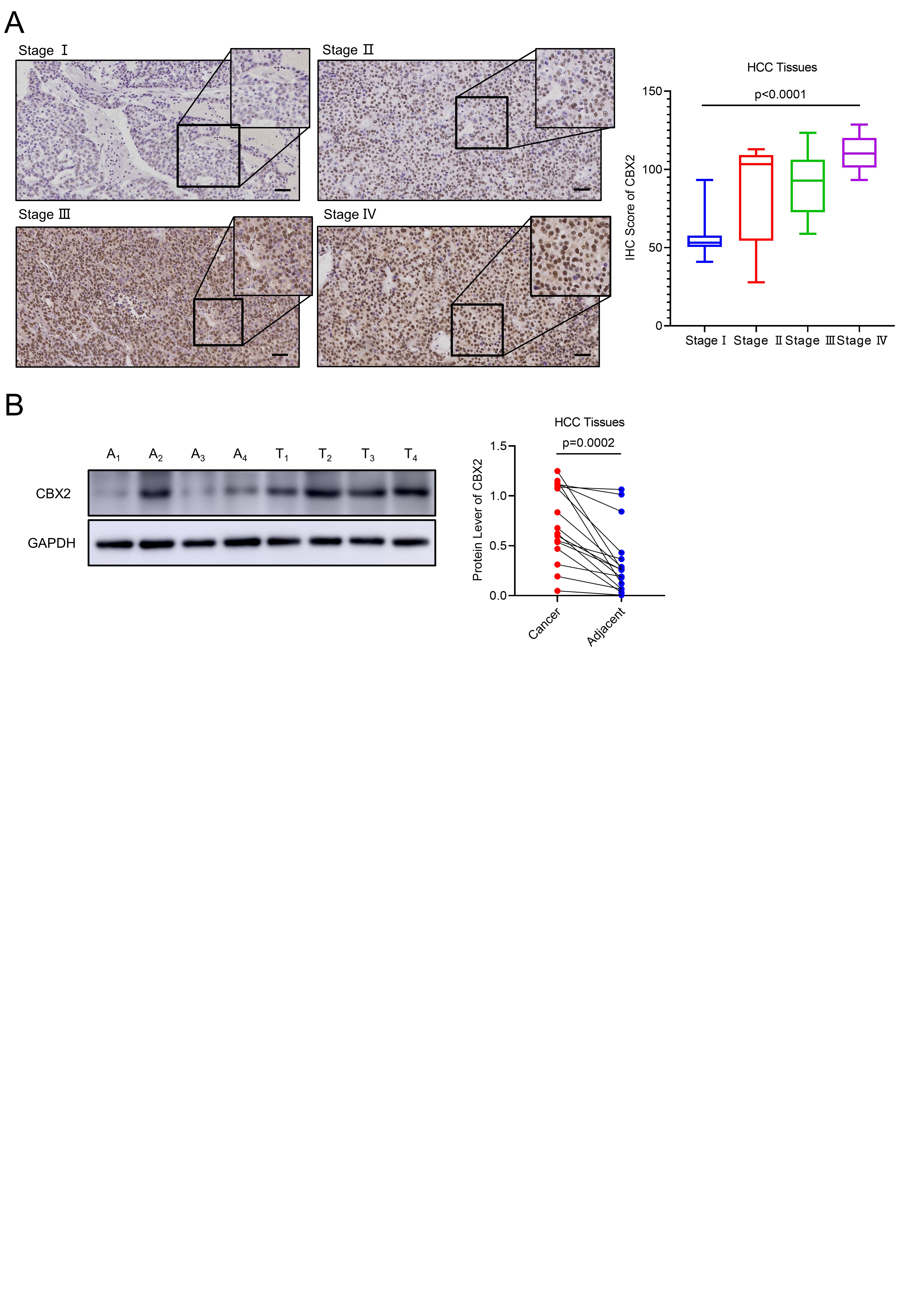

Supplement: Supplementary file 5 — Additional file 5. Fig. S4 The CBX2 is upregulated in HCC tissues. A. The immunohistochemistry assay of different stage HCC tissues. N=13 for Stage I, N=13 for Stage II, N=16 for Stage III and N=14 for Stage IV. The black bar represents 100μm. B. The representative immunoblots of CBX2 in tumor (T1~T4) and adjacent (A1~A4) tissue of HCC patients. The semiquantitative analysis were performed by integrated optical density measurement with Image J software. [file 13148_2022_1393_MOESM5_ESM.jpg]

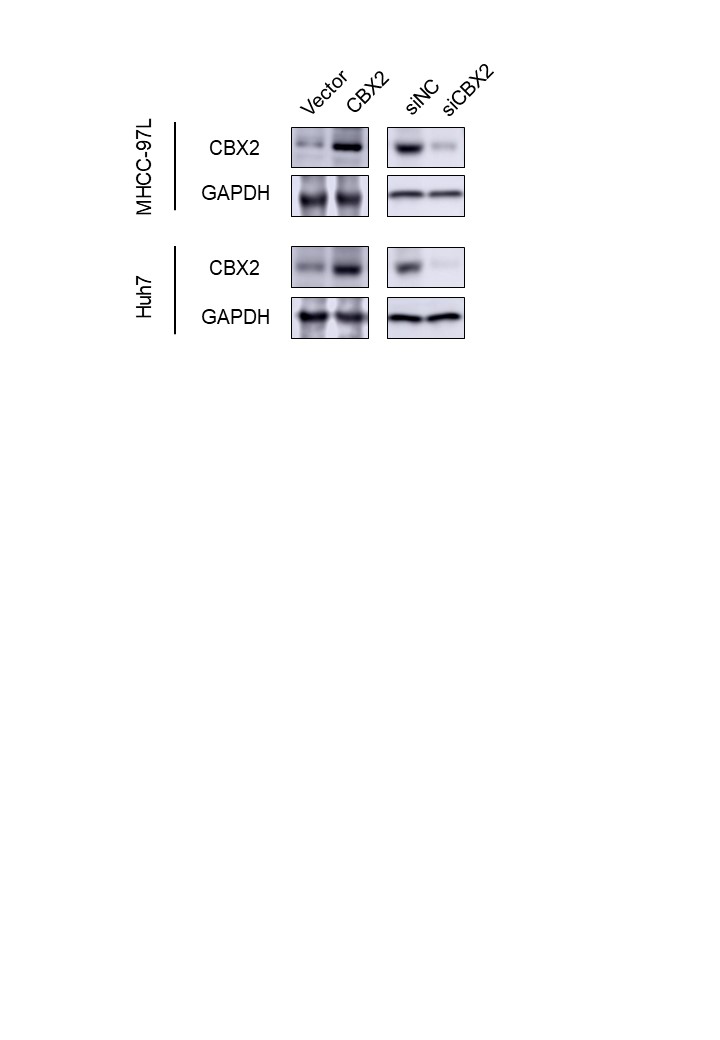

Supplement: Supplementary file 6 — Additional file 6. Fig. S5 The CBX2 protein expression after knockdown and overexpression in HCC cells. [file 13148_2022_1393_MOESM6_ESM.jpg]
